# Percutaneous Thrombectomy of Clot in Transit Lodged in the Patent Foramen Ovale Using the FlowTriever Device: Case Report

**DOI:** 10.1016/j.jscai.2025.103879

**Published:** 2025-08-28

**Authors:** Sachin Joseph, Pablo Rengifo-Moreno

**Affiliations:** a Internal Medicine, FSU College of Medicine, Tallahassee, Florida; b Interventional and Structural Cardiology, Tallahassee Memorial Hospital, Tallahassee, Florida

**Keywords:** case report, clot in transit, impending paradoxical embolism, sentinel device

## Abstract

A clot in transit is a venous thrombus lodged in the right heart en route to the pulmonary arteries. When a clot in transit lodges in a patent foramen ovale, it becomes an impending paradoxical embolism (IPDE), a rare, life-threatening condition with a 30-day mortality of 18.4%. Management options—surgical extraction, anticoagulation, or thrombolysis—lack consensus. We present the case of a 53-year-old man with a saddle pulmonary embolism, in whom postthrombectomy transthoracic echocardiogram revealed an IPDE. This case illustrates aspiration thrombectomy of the IPDE with the FlowTriever device (Inari Medical) with cerebral protection (SENTINEL, Boston Scientific), offering an alternative to thrombolysis and surgery.

## Introduction

Clot in transit (CIT) is a venous thrombus temporarily lodged in the right heart before entering the pulmonary vasculature. The CIT is rare, with an incidence of 4% to 18% in patients with pulmonary embolism.^1^ Often, CIT can be located in the right atrium, right ventricle, superior vena cava, or septal defects. Patent foramen ovale (PFO) is a common, often asymptomatic congenital heart defect, seen in 27% of the population.^2^ Impending paradoxical embolism (IPDE) is the presence of thrombus in the PFO. Paradoxical embolism makes up <2% of all arterial emboli. The IPDE is an even rarer diagnosis.^3^ We describe the case of a 53-year-old man who underwent aspiration thrombectomy of an IPDE using the FlowTriever device (Inari Medical) with concomitant cerebral protection via the SENTINEL device (Boston Scientific).

## Case presentation

A 53-year-old man with hypertension and a history of right tibial plateau fracture managed with knee immobilization 1 month before presented to an outside facility with 2 hours of shortness of breath and chest tightness. The patient reported being mostly sedentary since his injury. His vitals were notable for sinus tachycardia at 118 beats per minute, blood pressure was 114/87 mm Hg, and oxygen saturation was 89% on room air, which improved to 96% on nonrebreather at 15 L/min, with a respiratory rate of 26 breaths per minute. His troponin was 26 ng/L, pro–B-type natriuretic peptide was 1324 ng/L.

Given the clinical suspicion for pulmonary embolism (PE), a computed tomography (CT) pulmonary angiography was ordered, which showed a saddle embolus with acute bilateral pulmonary embolism, elevated right ventricular–to–left ventricular ratio of 2.2 consistent with acute right heart strain. Bilateral Doppler ultrasonography revealed a right popliteal vein deep venous thrombosis. The patient underwent successful mechanical thrombectomy of the PE via the right common femoral vein. A postthrombectomy transthoracic echocardiogram showed a PFO measuring 1.2 cm with a large mobile echocardiogram density, which was consistent with IPDE attached to the interatrial septum measuring 5.0 × 1.8 cm in the right atrial side and 1.8 × 0.7 cm in the left atrium. The patient was anticoagulated with heparin and transferred to our facility for further management of intracardiac thrombus.

**Figure 1:**
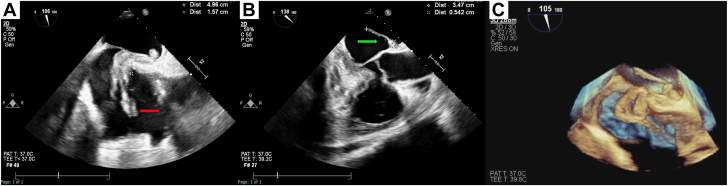
**Transesophageal echocardiogram (TEE) images.** (A) TEE showing thrombus in the right atrium (red arrow) crossing the PFO and extending into the left atrium. (B) TEE showing thrombus in the left atrium (green arrow). (C) Three-dimensional TEE showing clot in transit lodged in the patent foramen ovale.

**Figure 2:**
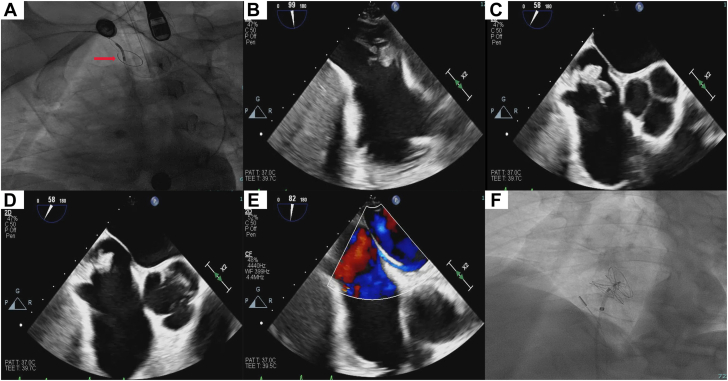
**Periprocedural imaging.** (A) Fluoroscopic view showing the SENTINEL device (red arrow). (B) Transesophageal echocardiogram (TEE) thrombus in contact with the FlowTriever. (C) TEE showing FlowTriever suctioning the thrombus. (D) TEE showing thrombus adherent to the tip of the FlowTriever device in a lollipop-like configuration. (E) TEE showing empty patent foramen ovale (PFO) with color Doppler flow across the PFO. (F) Fluoroscopic view showing PFO closure with 30.0-mm GORE CARDIOFORM Septal Occluder.

On arrival, the patient developed transient slurred speech concerning for a new neurological event. A CT angiography of the head and neck and a noncontrast CT head, both performed before the IPDE thrombectomy, showed no large vessel occlusion or acute intracranial abnormalities. His symptoms improved after 30 minutes. During the heart team discussion, cardiothoracic surgery expressed concern about operating on a patient that just had a transient ischemic attack and a PE, and recommended a percutaneous approach. After discussing the findings and the possible therapeutic options, it was decided to move forward with percutaneous mechanical thrombectomy along with cerebral embolic protection. The procedure was performed under general anesthesia and transesophageal echocardiographic (TEE) monitoring (Figure 1 and Supplemental Video 1 and 2). To minimize the risk of thrombus embolization, we avoided excessive manipulation in the right atrium and did not use intracardiac echocardiography. Using right radial access, the SENTINEL Cerebral Protection System (Boston Scientific) was inserted in the right innominate artery and left common carotid artery (Figure 2A, Supplemental Video 6). We obtained ultrasound-guided and fluoroscopy-guided access to the left femoral vein using the micropuncture technique. The access was secured using a 7F sheath. The venous access was dilated using a 22F dilater. Over a stiff wire, a 26F sheath was exchanged (Inari Medical). A 24F Triever catheter (Inari Medical) was advanced under fluoroscopy and TEE guidance in order to be close to the emboli in the right atrium. By inserting a curved T20 catheter inside a T24 and advancing it so the T20 adopted a steerable curve, we were able to precisely manipulate and position the system near the emboli before initiating suction (Figure 2B). Once we were close to the emboli, we performed our first aspiration (Figure 2C, Supplemental Video 3). There was partial return of blood with no thrombus in the filter. A second aspiration yielded the same result. At this point, the catheter was removed. The catheter had the clot at the tip in a “lollipop” fashion (Figure 2D, Supplemental Video 4). The sheath was also aspirated with no evidence of emboli. Postprocedural TEE confirmed no residual IPDE (Figure 2E, Supplemental Video 5), and the sentinel device was removed without evidence of emboli in the filters. Subsequently, the patient underwent successful transcatheter PFO closure with a 30.0-mm GORE CARDIOFORM Septal Occluder without complications (Figure 2F). He was discharged home on apixaban and clopidogrel.

## Discussion

The first case of IPDE was published by Nellessen et al^4^ in 1985. Advances in diagnostic imaging including thrombectomy transthoracic echocardiogram and TEE have led to increased reporting of this condition; however, there still remains a paucity of data due to the rarity of IPDE.

Impending paradoxical embolism is associated with a mortality of 18% with two-thirds of the deaths occurring within the first 24 hours after diagnosis, largely due to cardiogenic shock and right heart failure.^5^ Several treatment options including thrombolysis, surgical thrombectomy, percutaneous thrombectomy, and anticoagulation have been proposed, although there is currently no evidence to support superiority of one treatment over the other. The use of systemic anticoagulation alone for treatment of IPDE has been associated with an almost 35% mortality rate.^6^ Systemic anticoagulation is usually reserved for patients with major contraindication to surgery or fibrinolysis. Thrombolysis was associated with systemic embolism in 23.5% of patients.^5^ Surgical thrombectomy was associated with nonsignificant decreased odds of systemic embolism compared with anticoagulation alone. Baydoun et al^7^ proposed an algorithm for managing IPDE based on hemodynamic status and surgical risk. Hemodynamically stable, low-to-moderate surgical risk patients are recommended for emergent surgery, whereas anticoagulation was advised for stable patients with high surgical risk. In unstable patients, thrombolysis was the preferred approach, or anticoagulation if thrombolysis was not feasible. However, evidence seems to be scarce for the role of percutaneous thrombectomy for IPDE.

Piliero et al^8^ described a case of successful percutaneous removal of an IPDE using the FlowTriever device. Originally approved by the Food and Drug Administration in 2018 for intermediate-risk PE, the FlowTriever system has demonstrated significant improvement in right ventricular–to–left ventricular ratios with an excellent safety profile.^9^ Minimally invasive options for removing tricuspid vegetations and right-sided cardiac masses in high-risk surgical patients without the need for cardiopulmonary bypass include FlowTriever, AngioVac (AngioDynamics), AlphaVac (AngioDynamics), Lightning Flash 2.0 (Penumbra), Symphony (Imperative Care), and ŌNŌ (B. Braun), with additional devices currently in the experimental phase.^10^ PFO closure is typically done in patients with IPDE to avoid recurrent events. Since the patient had a tibial fracture and the venous thromboembolism was provoked, he was discharged on apixaban for 6 months. We decided to protect him long-term with PFO closure. Clopidogrel was also added to his discharge medications owing to a risk of thrombus formation on the PFO closure device within the first 3 months of device placement.

In this case, a SENTINEL Cerebral Protection System was used to prevent paradoxical embolization during the procedure, ensuring temporary protection of the cerebral circulation. The successful removal of the IPDE using a minimally invasive approach highlights the potential of aspiration thrombectomy as an alternative to traditional surgical options, which often carry significant morbidity. This case provides valuable insights into the management of high-risk patients and supports the use of innovative strategies for treating similar presentations.
